# Garlic: Much More Than a Common Spice

**DOI:** 10.3390/foods9111544

**Published:** 2020-10-26

**Authors:** Michael E. Netzel

**Affiliations:** Queensland Alliance for Agriculture and Food Innovation (QAAFI), The University of Queensland, Coopers Plains, QSL 4108, Australia; m.netzel@uq.edu.au; Tel.: +61-7-344-32476

**Keywords:** garlic, *Allium sativum* L., phytochemicals, organosulfur compounds, bioactivity, health benefits

## Abstract

Garlic is a widely consumed and popular spice with a characteristic “aroma” or odour. It contains a broad range of bioactive components such as organosulfur compounds, saponins and polyphenols, but can be also rich in vitamins and minerals. Numerous biological properties are attributed to garlic, from antimicrobial activities to neuro- and renal-protection. In addition, post-harvest treatment, storage and processing, such as fermentation and heat, can have a significant effect on garlic and its bioactive compounds, and subsequently alter its bioactive properties. Future studies are warranted to elucidate the “full” biological potential of garlic including well designed human clinical trials, detailed storage and processing studies as well as sophisticated in vitro cell culture models to better understand the underlying mechanisms of action.

Garlic (*Allium sativum* L.) has been known as the “aroma” vegetable, which is widely used as a food ingredient in many countries and different cultures as a result of its characteristic flavour and potential health benefits. Many studies have shown evidence of a significant reduction in the risk of developing chronic diseases (e.g., cardiovascular, cancer, obesity, diabetes, high blood pressure) associated with garlic consumption [1,2,3,4]. Together with therapeutic functions, garlic possesses additional biological activities such as antibacterial, antifungal, and antioxidant properties [5,6,7], resulting in garlic being one of the most important vegetables/spices worldwide [8]. It has been suggested that the biological properties and health benefits of garlic are derived from its phytochemicals, mainly organosulfur compounds, saponins and polyphenols.

Garlic possesses γ-glutamyl-S-alk(en)yl-l-cysteines and S-alk(en)yl-l-cysteine sulfoxides, particularly l-alliin as the major sulfur-containing compound in intact garlic [1]. Under different physical treatments (e.g., cutting, crushing, or chewing), the enzyme alliinase, released from the vacuole, lyses the S-alk(en)yl-l-cysteine sulfoxides to liberate the majority of the characteristic aroma thiosulfinate compounds such as allicin, diallyl sulfide, and diallyl disulfides [9,10]. These volatile compounds are extremely unstable and rapidly decompose to form other sulfur-containing compounds, which might not be the genuinely active compounds of garlic [11]. In general, organosulfur compounds in raw garlic have higher digestibility than those in cooked garlic [12]. The total amount of saponins and the saponin profile can vary considerably between different garlic genotypes, e.g., purple garlic contained almost 40 times more saponin than its white counterpart, and several saponin compounds could only be found in purple genotypes, such as desgalactotigonin-rhamnose, proto-desgalactotigonin, proto-desgalactotigonin- rhamnose, voghieroside D1, sativoside B1-rhamnose, and sativoside R1 [1]. In addition to organosulfur compounds, garlic contains a diverse range of phenolic compounds such as phenolic acids [13,14,15], quercetin [16] and anthocyanins [5]. Whilst organosulfur compounds are extremely unstable and susceptible to further transformation, recent attention has been placed on polyphenols due to their potential role in health-related benefits for humans [5]. Apart from its organosulfur compounds, saponins and polyphenols, garlic is also rich in vitamins and minerals [17]. However, it should be noted that the content of these bioactive compounds can vary considerably depending on the genotype, agronomic conditions, environmental factors, maturity, and post-harvest treatment [17,18,19].

A broad range of biological functions and properties are attributed to garlic, including antioxidant capacity, anti-inflammatory activity, antimicrobial activity, modulation of the immune system, cardiovascular protection, anticancer activity, hepatoprotective activity, protection of the digestive system, anti-diabetic activity, anti-obesity activity as well as neuro- and renal-protection. However, it should be noted that most of the reported biological and functional properties of garlic have been demonstrated in in vitro cell cultures or animal models, but only a few human trials have been conducted [20].

Based on the available literature, garlic and its bioactive compounds can be regarded as promising ingredients for functional food applications and nutraceuticals, but also as a “simple” spice in a diverse and healthy diet. In addition, the effects of post-harvest treatment, storage and processing, such as fermentation and heat, on garlic and its bioactive compounds should be further studied since this can have a significant impact on the biological properties and nutritional quality (unwanted degradation products/food safety) of this popular spice. Future studies are warranted to elucidate the “full” biological potential of garlic including well designed human clinical trials (bioavailability and intervention), detailed storage and processing studies as well as sophisticated in vitro cell culture models to gain a better understanding of the underlying mechanisms of action.

The review on bioactive compounds and biological functions of garlic by Shang et al. [20] gives a comprehensive overview of the reported biological activities and potential health benefits of garlic, its main bioactive compounds and provides a critical discussion of the postulated mechanisms of action.
